# Chronic conditions and medical expenditures among non-institutionalized adults in the United States

**DOI:** 10.1186/s12939-014-0105-3

**Published:** 2014-11-26

**Authors:** De-Chih Lee, Leiyu Shi, Geraldine Pierre, Jinsheng Zhu, Ruwei Hu

**Affiliations:** Department of Information Management, Da-Yeh University, Changhua, 51591 Taiwan; Department of Health Policy & Management, Bloomberg School of Public Health, Johns Hopkins University, Baltimore, Maryland 21205 USA; Johns Hopkins Primary Care Policy Center, Bloomberg School of Public Health, Johns Hopkins University, Baltimore, Maryland 21205 USA; School of Public Health and Center of Migrant Health Policy, Sun Yat-sen University, Guangzhou, 510080 P.R. China

**Keywords:** Chronic conditions, Health care expenditures, Vulnerable populations, MEPS

## Abstract

**Introduction:**

This study sought to examine medical expenditures among non-institutionalized adults in the United States with one or more chronic conditions.

**Method:**

Using data from the 2010 Medical Expenditure Panel Survey (MEPS) Household Component (HC), we explored total and out-of-pocket medical, hospital, physician office, and prescription drug expenditures for non-institutionalized adults 18 and older with and without chronic conditions. We examined relationships between expenditure differences and predisposing, enabling, and need factors using recent, nationally representative data.

**Results:**

Individuals with chronic conditions experienced higher total spending than those with no chronic conditions, even after controlling for confounding factors. This relationship persisted with age. Out-of-pocket spending trends mirrored total expenditure trends across health care categories. Additional population characteristics that were associated with high health care expenditures were race/ethnicity, marital status, insurance status, and education.

**Conclusions:**

The high costs associated with having one or more chronic conditions indicates a need for more robust interventions to target population groups who are most at risk.

## Introduction

Chronic disease stands as the leading cause of death and disability in the United States and most other countries in the world [1]. Seven out of ten deaths among Americans annually are from chronic disease, with heart disease, cancer and stroke accounting for more than 50 percent of these deaths [2]. In 2010, 21 percent of adults aged 45–64 and 45 percent of adults 65 and over had been diagnosed with two or more chronic conditions [3]. Research has shown a positive association between chronic disease and a number of factors, including poorer health-related quality of life and greater rates of depression and obesity [4-6]. More recently, there has been exploration into the relationship between chronic conditions and health care costs.

The Centers for Disease Control and Prevention (CDC) estimates that 75 percent of our health care dollars as a nation go to the treatment of chronic disease [1]. Estimates from researchers have revealed the costs of specific chronic diseases each year, including $432 billion on heart disease and stroke, $245 billion on diabetes, and $154 billion on lung disease [7-9]. A greater number of chronic conditions have been found to be associated with increased spending. The relationship between chronic disease and expenditures also appears to persist with age. In a 2011 American Journal of Managed Care publication, among a sample of adults 18–64 years of age, the mean medical cost per year for an individual with no chronic conditions was $2,137, while the cost for an individual with five or more conditions was $21,183. In the same study, the mean annual cost per person increased from $1,700 to $2,000 per additional chronic condition for enrollees with 0 to 4 chronic conditions [10]. A 2002 publication by Wolff and colleagues found that per capital Medicare expenditures increased with the number of chronic conditions, ranging from $211 among beneficiaries without a chronic condition to $13,973 among beneficiaries with 4 or more types of chronic conditions [11].

### New contribution

This research examines the cost of chronic conditions in the United States by looking at recent, nationally representative medical expenditure data from the 2010 Medical Expenditure Panel Survey (MEPS). This research presents an overall picture of spending associated with chronic conditions in the US, while considering predisposing, enabling, and need factors that are associated with spending. The study looks at both mid-aged and older adults, whereas previous studies have focused only on certain age subgroups. In addition, this research considers overall expenditures associated with having one or more chronic conditions, rather than expenditures associated with an incremental number of conditions. For example, a study by Naessens and colleagues examined the longitudinal effect on health care costs of having 0, 1, 2, 3, 4, or 5 or more chronic conditions among adults 18 to 64 years of age using 2004 to 2007 data [10]. A 2002 study by Wolff et al. considered expenditures among Medicare fee-for-service beneficiaries aged 65 and older with 0, 1, 2, 3, or 4 or more chronic conditions [11].

This study considers total and out-of-pocket spending attributable to having chronic conditions among all adults in comparison to having no chronic conditions, in an effort to highlight the big picture and support the hypothesis that total medical expenditures, including hospital, physician office, and prescription drug costs, are higher among individuals with chronic conditions compared to those with no chronic conditions. Results of the study would further emphasize the need for equitable health policy to target care provision for people with chronic conditions across the life course. This is crucial, as additional resources and support may be necessary to ensure that this vulnerable group has access to affordable, appropriate, and adequate health care. It has been reported that the unique needs of vulnerable populations such as those with chronic conditions have not been adequately reflected in local planning, policy/decision making and service provision [12]. Concerted efforts to fight chronic diseases can advance health equity and development, both nationally and globally [13].

## Methods

### Data

The Household Component (HC) of the 2010 US Medical Expenditure Panel Survey (MEPS) was used for this study. There were 32,846 un-weighted observations in the dataset. MEPS is a nationally representative survey of the US noninstitutionalized civilian population, conducted by the Agency for Healthcare Research and Quality (AHRQ). Each annual survey is a nationally representative subsample of households, based on the sampling frame of the prior year’s National Health Interview Survey (NHIS), which uses a stratified, multistate sampling design. The survey uses an overlapping panel design, where data are collected over a 2 ½ year period. MEPS is unique in its ability to link data on individuals and households to information on health services use and expenditures. Additional information regarding MEPS has been described elsewhere [14].

### Measures

The household component of MEPS includes information collected from individual household members and their medical providers on demographic characteristics, health conditions, health status, use of medical care services, charges and payments, access to primary care, satisfaction with care, health insurance coverage, income, and employment [15]. For this particular study, the dependent variables of interest are total and out-of-pocket expenditures related to total medical care, as well as hospital use, physician office visits, and prescription drug use. Expenditures data were obtained through medical provider documentation.

Aday and Andersen’s access to care framework was used in the selection of covariates that may be related to total and out-of-pocket expenditures [16-18]. Covariates (independent variables) of interest in the study were considered to be predisposing factors, enabling factors, or need factors. Predisposing factors that were included are age, sex, race/ethnicity, health insurance, highest education degree, employment status, and marital status. In addition, having a chronic condition was considered to be a predisposing factor in our analysis. Enabling factors were household income, provider type of usual source of care (USC), Metropolitan Statistical Area (MSA) residence, and Census region. Need factors included in our analysis were perceived health status, perceived mental health status, IADL help screener, and ADL help screener.

Predisposing factors were represented as either binary or categorical variables. Having a chronic condition was measured dichotomously, with no chronic conditions serving as the reference category. Age was measured as 18–64 years of age or above 64 years of age (reference). Sex was measured as male or female (reference). Race/ethnicity was represented categorically, with individuals categorized as non-Hispanic white (reference), non-Hispanic black, Hispanic, non-Hispanic Asian, or other. Health insurance categories were private (reference), public, or no insurance. Education was a categorical variable, measured by no degree (reference), high school diploma, Bachelor’s degree and above, or other degree. Employment status categories were not employed, employed, or inapplicable. Marital status categories were not married, married, or inapplicable.

Enabling factors were measured as categorical variables. Household income categories were less than $20,000 (reference), $20,000-$39,999, and greater than $40,000. Provider type of usual source of care (USC) was reported as a facility (reference), person, or person within a facility. Census region was measured as Northeast, Midwest, South (reference), or West. Residence in a Metropolitan Statistical Area (MSA) was measured dichotomously, with non-MSA residence serving as the reference. Need factors were measured similarly. Perceived health status and perceived mental health status were reported as fair/poor (reference) or excellent/very good/good. Use of an Instrumental Activity of Daily Living (IADL) help screener and Activity of Daily Living (ADL) help screener were measured dichotomously, with no screener use serving as the reference category.

### Statistical analysis

Data analysis was performed using SAS Version 9.3. Analysis of Variance (ANOVA) tests were used to test the significance of differences within variables. Multivariate regression was used to estimate medical expenditures for the population of interest. Due to the complex sampling of MEPS, all analyses accounted for the design effect and the sampling weights.

## Results

Table 1 displays weighted population characteristics for individuals with and without chronic conditions. A total of 114,372,238 individuals with chronic conditions and 116,770,853 individuals without chronic conditions were represented. With the exception of income, statistically significant differences existed between the chronic condition and no chronic condition groups across all predisposing, enabling, and need factors (p < .001).

**Table 1:** **Summary of population characteristics by chronic condition status (weighted frequency and percentage)**

	**Without chronic conditions (%)**	**With chronic conditions (%)**
**Predisposing factors**		
Age in years***		
18-64	110,150,360 (96.31)	81,181,397 (69.52)
Above 64	4,221,878 (3.69)	35,589,456 (30.48)
Sex***		
Male	57,312,421 (50.11)	54,650,247 (46.8)
Female	57,059,817 (49.89)	62,120,606 (53.2)
Race/Ethnicity***		
Non-Hispanic White	71,882,464 (62.85)	84,417,517 (72.29)
Non-Hispanic Black	12,034,849 (10.52)	14,640,660 (12.54)
Hispanic	20,948,350 (18.32)	11,671,261 (10)
Non-Hispanic Asian	7,199,312 (6.29)	3,715,578 (3.18)
Others	2,307,263 (2.02)	2,325,837 (1.99)
Health insurance***		
Private	79,372,453 (69.4)	76,817,936 (65.79)
Public	11,316,674 (9.89)	28,292,792 (24.23)
No insurance	23,683,111 (20.71)	11,660,125 (9.99)
Highest education degree***		
No degree	17,455,057 (15.36)	16,853,562 (14.5)
High school diploma	53,040,465 (46.68)	57,802,675 (49.72)
Bachelor’s degree and above	34,130,802 (30.04)	30,673,666 (26.39)
Other degree	8,989,106 (7.91)	10,919,503 (9.39)
Employment status***		
Not employed	24,793,095 (21.77)	51,875,893 (44.53)
Employed	88,954,971 (78.12)	64,586,933 (55.44)
Inapplicable	118,200 (0.1)	41,275 (0.04)
Marital status***		
Not married	57,682,490 (50.43)	50,977,590 (43.66)
Married	56,571,548 (49.46)	65,751,988 (56.31)
Inapplicable	118,200 (0.1)	41,275 (0.04)
**Enabling factors**		
**Income**		
<$20,000	49,258,353 (43.13)	49,241,346 (42.22)
$20,000-$39,999	28,824,836 (25.24)	30,736,220 (26.35)
> = $40,000	36,121,612 (31.63)	36,651,553 (31.43)
Provider type of USC***		
Facility	41,857,172 (36.6)	46,300,960 (39.65)
Person	20,555,369 (17.97)	30,358,779 (26)
Person in facility	13,058,326 (11.42)	22,969,617 (19.67)
Inapplicable/DK/Refused/Not Ascertained	38,901,370 (34.01)	17,141,496 (14.68)
MSA***		
No	15,238,959 (13.32)	21,183,918 (18.14)
Yes	99,133,279 (86.68)	9,558,6935 (81.86)
Census region***		
Northeast	21,183,642 (18.52)	21,464,981 (18.38)
Midwest	23,255,565 (20.33)	26,946,032 (23.08)
South	41,028,577 (35.87)	43,760,170 (37.48)
West	28,904,455 (25.27)	24,599,670 (21.07)
**Need factors**		
Perceived health status***		
Excellent/VG/Good	108,791,996 (95.27)	92,586,538 (79.35)
Fair/Poor	5,404,615 (4.73)	24,100,352 (20.65)
Perceived mental health status***		
Excellent/VG/Good	109,721,106 (96.08)	104,068,620 (89.22)
Fair/Poor	4,474,620 (3.92)	12,570,991 (10.78)
IADL help screener ***		
No	113,012,742 (99.12)	109,882,307 (94.17)
Yes	1,000,364 (0.88)	6,802,924 (5.83)
ADL help screener***		
No	113,461,189 (99.59)	113,331,567 (97.15)
Yes	461,586 (0.41)	3,329,220 (2.85)

***p <0.001.

Table 2 compares the weighted unadjusted means of total medical, hospital, physician office, and prescription drug expenditures by population characteristics. Among individuals with chronic conditions, adults age 18–64 were found to have a total mean medical expenditures of $5,946 compared to those above the age of 64, who experienced higher average total medical expenditures of $10,452 (p <0.001). This relationship was similar for hospital, physician office, and prescription drug expenditures (p <0.001). Females with chronic conditions experienced significantly higher prescription drug expenditures compared to males (p <0.001). Non-Hispanic whites had higher average medical, physician office, and prescription drug expenditures compared to non-Hispanic Blacks, Hispanics, non-Hispanic Asians, and others. Conversely, non-Hispanic blacks and individuals of other race/ethnicity reported higher mean hospital expenditures than whites, Hispanics, and Asians. Individuals with chronic conditions with public health insurance experienced greater average expenditures than those with private insurance and the uninsured in every category. When considering education, those with a Bachelor’s degree or above had higher physician office expenditures than those with no degree or a high school degree. Those with other degrees reported highest average spending on physician office visits. The unemployed experienced greater expenditures than employed individuals across all categories. In addition, married persons spent more than those who were not married on hospital costs and physician office visit, and spent less on hospital stays and prescription drugs.

**Table 2:** **Population characteristics and medical expenditures for those with and without chronic conditions**

		**Without chronic conditions (n =114,372,238)**				**With chronic conditions (n =116,770,853)**			
	**Population**	**Total medical expenditure**	**Total hospital expenditure**	**Total physician office expenditure**	**Total prescription drug expenditure**	**Total medical expenditure**	**Total hospital expenditure**	**Total physician office expenditure**	**Total prescription drug expenditure**
**Predisposing factors**									
Age in years		*		**	***	***	***	***	***
18-64	191,331,757	2134.29 (92.30)	879.32 (67.13)	414.90 (23.20)	349.77 (30.20)	5946.07 (192.70)	2684.16 (160.13)	925.26 (35.85)	1487.41 (46.44)
Above 64	39,811,334	3387.65 (521.73)	1191.20 (386.49)	715.01 (93.64)	803.23 (116.69)	10451.61 (329.53)	4334.02 (241.36)	1700.30 (108.97)	2504.21 (79.66)
Sex		***	***	***	*				***
Male	111,962,667	1554.40 (110.48)	548.30 (73.80)	269.77 (21.40)	308.05 (42.13)	7022.29 (266.04)	3237.63 (221.39)	1107.52 (75.81)	1646.84 (57.59)
Female	119,180,423	2809.49 (138.30)	1234.89 (110.81)	582.88 (36.26)	425.22 (39.45)	7580.53 (226.23)	3142.47 (179.70)	1208.94 (38.13)	1929.68 (52.77)
Race/Ethnicity		***	*	***	***	***	***	**	***
Non-Hispanic White	156,299,981	2553.94 (130.07)	995.96 (99.29)	516.16 (34.22)	447.93 (31.76)	7723.10 (205.76)	3306.59 (164.60)	1235.98 (50.05)	1919.35 (49.79)
Non-Hispanic Black	26,675,509	1850.14 (199.12)	921.97 (131.01)	284.58 (38.64)	219.05 (74.37)	7175.68 (451.64)	3522.66 (339.88)	980.28 (85.61)	1588.27 (99.70)
Hispanic	32,619,611	1403.18 (144.04)	634.15 (81.72)	256.39 (24.62)	257.92 (91.38)	5357.97 (342.07)	2196.44 (227.77)	937.47 (97.78)	1386.71 (132.83)
Non-Hispanic Asian	10,914,890	1509.82 (199.03)	567.62 (155.82)	333.06 (43.67)	172.29 (39.46)	5219.55 (588.44)	2137.29 (451.44)	1037.86 (227.81)	1254.18 (151.07)
Others	4,633,100	1422.08 (348.96)	792.30 (329.57)	183.49 (28.78)	190.81 (76.99)	6762.09 (1312.87)	3381.44 (1177.12)	919.38 (124.17)	1611.56 (228.35)
Health insurance		***	***	***	***	***	***	***	***
Private	156,190,389	2493.83 (118.24)	1012.03 (90.04)	510.08 (30.04)	383.87 (29.96)	7036.46 (212.70)	2964.13 (183.19)	1237.28 (53.64)	1692.69 (47.07)
Public	39,609,466	3107.28 (296.86)	1136.05 (123.60)	499.53 (65.18)	875.27 (187.74)	9978.46 (424.06)	4488.11 (282.83)	1286.26 (65.72)	2580.35 (109.89)
No insurance	35,343,236	687.81 (104.69)	367.48 (90.33)	108.98 (16.55)	65.21 (8.26)	2730.04 (301.15)	1498.29 (260.35)	359.25 (37.47)	586.56 (51.31)
Highest education degree		***	*	***	**			*	
No degree	34,308,619	1448.60 (137.10)	552.96 (80.35)	239.01 (20.41)	237.74 (41.61)	7349.24 (390.39)	3422.86 (293.94)	933.54 (91.23)	1862.32 (106.90)
High school diploma	110,843,140	1930.35 (126.05)	844.26 (87.00)	357.99 (26.68)	320.74 (46.11)	7181.19 (217.76)	3020.60 (152.26)	1178.33 (67.74)	1813.58 (54.43)
Bachelor’s degree and above	64,804,468	2888.13 (195.73)	1082.24 (147.29)	638.00 (58.78)	490.11 (57.10)	7511.46 (404.74)	3204.76 (350.68)	1232.89 (62.68)	1793.23 (78.50)
Other degree	19,908,609	2482.64 (429.98)	1128.84 (361.39)	403.29 (48.80)	436.43 (109.25)	7473.49 (597.37)	3574.69 (519.50)	1257.15 (120.12)	1647.59 (131.66)
Employment status		***	***	***	**	***	***	***	***
Not employed	76,668,988	2785.99 (258.90)	1229.54 (189.98)	431.58 (27.11)	644.59 (101.25)	9833.12 (271.83)	4235.89 (207.36)	1503.20 (77.37)	2548.12 (74.46)
Employed	153,541,903	2020.95 (90.06)	800.77 (65.86)	426.18 (27.54)	289.59 (24.78)	5329.62 (222.37)	2358.57 (177.99)	891.76 (37.55)	1201.30 (41.18)
Inapplicable	159,475	550.27 (374.47)	49.09 (51.02)	70.40 (34.30)	386.24 (368.30)	51.56 (30.81)	0.00 (0.00)	0.00 (0.00)	51.56 (30.81)
Marital status		***	***	***		***	***	***	***
Not married	108,660,080	1762.24 (97.52)	669.79 (66.63)	318.04 (20.09)	329.73 (44.56)	7343.67 (248.93)	3068.81 (157.80)	1136.41 (52.90)	1817.11 (63.36)
Married	122,323,536	2610.49 (141.83)	1117.98 (109.94)	536.78 (39.84)	403.97 (36.46)	7304.91 (248.46)	3280.64 (210.59)	1181.64 (59.97)	1783.05 (52.38)
Inapplicable	159,475	550.27 (374.47)	49.09 (51.02)	70.40 (34.30)	386.24 (368.30)	51.56 (30.81)	0.00 (0.00)	0.00 (0.00)	51.56 (30.81)
**Enabling factors**									
**Income**				***		***	***		***
<$20,000	98,499,699	2059.97 (146.42)	961.52 (123.80)	347.53 (22.84)	335.83 (32.19)	8198.68 (284.53)	3829.06 (224.85)	1140.64 (56.42)	2059.75 (70.84)
$20,000-$39,999	59,561,056	2101.75 (170.05)	920.19 (121.11)	374.74 (36.82)	361.02 (73.93)	6455.36 (266.18)	2513.21 (180.08)	1182.44 (107.88)	1672.23 (67.36)
> = $40,000	72,773,165	2389.94 (114.42)	764.87 (65.28)	574.26 (53.67)	413.32 (50.43)	6856.75 (313.49)	2886.41 (240.69)	1174.15 (61.41)	1551.77 (56.79)
Provider type of USC		***	**	***	***	***	*	***	***
Facility	88,158,133	2515.29 (136.86)	935.28 (92.14)	504.77 (36.89)	452.32 (39.29)	7472.14 (275.30)	3188.66 (210.94)	1261.60 (80.76)	1829.53 (69.46)
Person	50,914,148	2771.93 (241.33)	1109.57 (180.93)	577.76 (76.96)	521.17 (90.59)	7847.49 (316.39)	3333.67 (264.16)	1252.32 (64.54)	2008.32 (74.15)
Person in facility	36,027,944	3387.82 (360.96)	1455.24 (260.06)	584.92 (53.64)	650.36 (146.46)	8922.97 (477.85)	3774.09 (318.75)	1352.02 (74.20)	2267.35 (92.76)
Inapplicable/DK/Refused/Not Ascertained	56,042,866	1102.66 (118.76)	537.98 (98.40)	207.66 (24.77)	97.18 (13.06)	3821.84 (513.22)	2136.09 (467.09)	474.79 (73.03)	706.69 (86.31)
MSA									
No	36,422,877	2211.18 (232.57)	961.76 (162.43)	380.76 (40.10)	435.55 (100.70)	7279.21 (391.81)	3113.89 (277.72)	1119.95 (66.82)	1892.12 (96.75)
Yes	194,720,214	2175.85 (99.11)	879.93 (73.02)	432.93 (25.58)	355.90 (30.64)	7328.14 (191.48)	3203.21 (147.88)	1170.68 (48.44)	1776.30 (49.18)
Census region						*	**		*
Northeast	42,648,623	2687.87 (314.80)	1138.11 (203.83)	501.29 (48.73)	507.40 (114.35)	7468.72 (397.84)	3312.15 (346.44)	1203.72 (69.11)	1715.69 (79.86)
Midwest	50,201,597	2059.51 (130.60)	717.69 (66.69)	448.07 (58.08)	326.18 (42.52)	8363.83 (402.06)	4028.56 (339.69)	1214.89 (63.21)	1903.12 (85.80)
South	84,788,746	2057.88 (143.92)	874.00 (117.86)	386.70 (40.54)	380.87 (37.48)	6906.83 (271.74)	2969.69 (205.94)	1130.30 (86.83)	1871.65 (74.42)
West	53,504,125	2080.28 (151.09)	872.81 (116.11)	408.76 (36.64)	275.31 (45.39)	6778.35 (359.35)	2542.57 (239.76)	1121.56 (55.70)	1620.38 (76.97)
**Need factors**									
Perceived health status		***	***	***	***	***	***	***	***
Excellent/VG/Good	201,378,534	1897.68 (67.25)	715.83 (50.04)	400.71 (21.80)	297.39 (20.68)	5612.94 (170.98)	2201.59 (129.83)	985.41 (43.64)	1449.64 (35.54)
Fair/Poor	29,504,968	7929.34 (202.23)	4441.51 (882.45)	945.26 (132.12)	1760.98 (423.70)	13987 (541.60)	6983.80 (426.99)	1841.43 (98.25)	3138.51 (136.17)
Perceived mental health status		***	*	**	***	***	***	***	***
Excellent/VG/Good	213,789,726	2011.05 (82.17)	802.37 (54.70)	411.94 (22.73)	320.48 (29.46)	6771.72 (167.84)	2901.15 (132.67)	1112.93 (42.77)	1652.50 (40.14)
Fair/Poor	17,045,611	6404.00 (1083.34)	3093.95 (923.32)	783.50 (140.63)	1499.36 (213.76)	11865 (594.43)	5555.63 (509.46)	1572.05 (120.76)	2991.81 (143.96)
IADL help screener		**		*	**	***	***	***	***
No	222,895,048	2089.00 (86.22)	854.74 (63.43)	424.46 (22.81)	341.17 (27.54)	6447.33 (153.36)	2718.30 (122.32)	1115.62 (39.99)	1647.34 (39.47)
Yes	7,803,288	12352 (3089.00)	4570.58 (1888.42)	667.67 (117.00)	3289.24 (1119.26)	21483 (1319.09)	10793 (1113.48)	1916.16 (180.62)	4240.08 (284.56)
ADL help screener		**				***	***	**	***
No	226,792,756	2116.28 (87.91)	862.38 (64.26)	425.30 (22.70)	357.11 (29.08)	6792.16 (157.95)	2897.97 (125.28)	1138.09 (40.89)	1712.00 (40.15)
Yes	3,790,806	17565 (5347.54)	6887.16 (3361.57)	694.04 (207.65)	2858.11 (1631.82)	25454 (1980.99)	13113 (1743.15)	1988.03 (272.66)	4740.97 (394.85)

*p <0.05, **p <0.01, ***p <0.001.

Additionally, for those with chronic conditions, individuals in the lowest income, on average, experienced greater medical, hospital, and prescription drug expenditures. Individuals who reported a person within a facility as provider type of USC experienced higher spending than others in all categories. Individuals in the Midwest experienced greater medical, hospital, and prescription drug expenditures than those in any other region. Adults with chronic conditions who reported fair or poor health and mental health status spent significantly more than those who reported excellent, very good, or good status across total medical, hospital, physician office, and prescription drug expenditures. Also, use of an IADL or ADL help screener was associated with higher expenditures across all categories.

Among adults with no chronic conditions, similar associations that were smaller in magnitude existed between characteristics and mean expenditures, compared to those with chronic diseases. Older adults with no chronic conditions had higher medical, physician office, and prescription drug expenditures. Significantly higher spending was apparent for women compared to men across all categories. In considering race/ethnicity, non-Hispanic whites with no chronic conditions experienced significantly higher spending across all categories, compared to minorities. Individuals with public insurance had higher expenditures than the uninsured and those with private insurance on all measures, with the exception of physician office visits, in which privately insured patients had the highest expenditures. When looking at education, individuals with a Bachelor’s degree and above consistently had higher expenditures than others. Like those with chronic conditions, unemployed and married adults without chronic conditions experienced higher spending compared to those who were employed or not married. Individuals in the highest income group spent more than others only on physician office visits. Individuals without chronic conditions who reported a person in facility as their USC provider reported higher spending than others in all expenditure categories. Those who reported fair/poor physical and mental health status had higher spending across all categories than those with better self reported health status. Individuals with IADL and ADL help screeners experienced greater spending across all categories than those without screeners (p <0.05).

Similar results existed when considering total out-of-pocket medical care, hospital, physician office, and prescription drug expenditures by chronic condition status and population characteristics. Individuals with chronic conditions spent significantly more than those with no chronic conditions across several significant predisposing, enabling, and need factors. These results can be found in Table 3. ANOVA tests were used to test the differences within variables in both sets of analyses.

**Table 3:** **Population characteristics and out-of -pocket medical expenditures for those with and without chronic conditions**

		**Without chronic conditions**				**With chronic conditions**			
	**Population**	**Total out-of-pocket medical expenditure**	**Out-of-pocket hospital expenditure**	**Out-of-pocket physician office expenditure**	**Out-of-pocket prescription drug expenditure**	**Total out-of- pocket medical expenditure**	**Out-of-pocket hospital expenditure**	**Out-of-pocket physician office expenditure**	**Out-of-pocket prescription drug expenditure**
**Predisposing factors**									
Age in years		**	***		***	***			***
18-64	191,331,757	403.27 (15.59)	61.53 (5.16)	83.90 (7.68)	77.71 (6.19)	831.61 (23.58)	112.04 (8.96)	141.54 (8.29)	328.34 (10.51)
Above 64	39,811,334	686.38 (89.17)	18.77 (8.46)	87.11 (25.16)	200.17 (30.69)	1248.20 (41.56)	95.40 (16.29)	123.99 (9.52)	570.45 (17.75)
Sex		***	***	***	**	***		**	***
Male	111,962,667	326.54 (19.40)	43.25 (6.69)	54.83 (4.79)	65.24 (10.24)	861.39 (27.16)	109.69 (13.18)	117.36 (6.74)	361.70 (10.91)
Female	119,180,423	501.28 (22.76)	76.71 (6.90)	113.33 (12.86)	99.30 (6.93)	1044.08 (30.49)	104.58 (9.35)	152.77 (10.13)	437.70 (13.52)
Race/Ethnicity		***	***	***	***	***	**	***	***
Non-Hispanic White	156,299,981	528.34 (21.89)	72.21 (7.55)	107.73 (10.95)	112.91 (9.28)	1083.47 (27.15)	108.57 (8.03)	156.66 (8.94)	449.76 (11.65)
Non-Hispanic Black	26,675,509	152.95 (12.47)	32.27 (5.05)	31.43 (5.04)	22.35 (2.31)	614.53 (39.38)	115.49 (31.09)	70.08 (5.11)	283.06 (12.47)
Hispanic	32,619,611	234.71 (23.29)	45.63 (6.50)	49.25 (12.31)	33.78 (4.90)	580.53 (32.53)	70.46 (9.50)	86.69 (11.52)	252.76 (16.79)
Non-Hispanic Asian	10,914,890	305.44 (43.29)	32.22 (8.53)	54.78 (12.38)	32.93 (5.14)	669.41 (47.48)	66.77 (16.35)	91.02 (13.54)	275.93 (31.11)
Others	4,633,100	165.96 (39.44)	38.86 (24.81)	26.26 (5.49)	32.56 (8.03)	950.54 (186.25)	242.73 (145.89)	130.28 (46.03)	374.13 (73.62)
Health insurance		***	***	*	***	***		***	*
Private	156,190,389	485.88 (17.55)	71.20 (6.32)	95.92 (6.18)	92.77 (5.93)	1042.13 (29.84)	112.87 (9.89)	155.65 (8.52)	407.42 (11.85)
Public	39,609,466	293.71 (66.51)	17.73 (5.93)	65.36 (43.21)	106.45 (46.35)	838.50 (34.24)	82.74 (11.02)	91.06 (9.06)	423.33 (18.72)
No insurance	35,343,236	229.23 (21.22)	42.42 (8.72)	53.03 (13.80)	35.36 (3.89)	699.55 (49.24)	126.91 (22.44)	117.52 (17.77)	315.81 (31.38)
Highest education degree		***	***	***	***	***		***	**
No degree	34,308,619	204.30 (19.75)	31.46 (6.13)	33.14 (3.70)	46.73 (7.14)	651.50 (37.12)	95.28 (22.43)	66.95 (5.57)	360.52 (21.63)
High school diploma	110,843,140	368.71 (22.91)	55.37 (6.70)	71.71 (13.07)	73.59 (10.62)	931.03 (24.84)	100.62 (9.37)	117.34 (7.41)	414.69 (11.22)
Bachelor’s degree and above	64,804,468	600.68 (29.69)	82.58 (11.18)	132.89 (12.31)	116.70 (10.60)	1182.55 (51.08)	111.02 (12.90)	198.57 (16.75)	427.69 (21.09)
Other degree	19,908,609	394.50 (32.27)	59.69 (15.84)	73.88 (9.32)	75.17 (8.99)	973.31 (86.97)	147.88 (43.15)	171.48 (30.07)	338.05 (23.05)
Employment status		***	***		***	***	***	***	***
Not employed	76,668,988	434.85 (35.33)	65.08 (12.17)	76.37 (10.99)	112.39 (22.41)	1122.24 (34.42)	103.90 (12.48)	120.59 (8.74)	521.61 (15.75)
Employed	153,541,903	409.72 (15.93)	58.90 (5.37)	86.50 (8.73)	73.98 (5.16)	829.87 (22.38)	109.90 (9.90)	149.27 (9.16)	307.76 (8.66)
Inapplicable	159,475	96.73 (63.08)	0.00 (0.00)	49.54 (29.11)	5.91 (4.91)	37.34 (23.42)	0.00 (0.00)	0.00 (0.00)	37.34 (23.42)
Marital status		***	***	***	***	***	***	***	***
Not married	108,660,080	320.91 (17.06)	44.39 (5.76)	62.16 (9.65)	55.79 (3.48)	881.19 (29.02)	88.02 (9.76)	130.27 (10.14)	368.66 (13.57)
Married	122,323,536	509.01 (23.61)	75.93 (8.13)	106.38 (9.31)	109.35 (11.33)	1019.16 (29.21)	121.73 (11.78)	140.87 (7.94)	428.30 (12.96)
Inapplicable	159,475	96.73 (63.08)	0.00 (0.00)	49.54 (29.11)	5.91 (4.91)	37.34 (23.42)	0.00 (0.00)	0.00 (0.00)	37.34 (23.42)
**Enabling factors**									
**Income**		***		*		***		***	
<$20,000	98,499,699	347.87 (23.50)	60.29 (8.30)	74.54 (13.43)	73.04 (11.24)	884.10 (28.76)	101.61 (11.43)	107.39 (8.63)	424.43 (15.14)
$20,000-$39,999	59,561,056	388.28 (24.51)	59.33 (9.25)	71.73 (7.59)	75.22 (7.18)	938.53 (33.81)	113.80 (14.62)	130.92 (8.47)	391.55 (14.21)
> = $40,000	72,773,165	523.14 (27.91)	59.93 (7.40)	106.82 (11.07)	100.27 (9.95)	1076.52 (37.33)	108.72 (12.37)	179.43 (16.05)	380.86 (14.27)
Provider type of USC		***	**		***	***		***	***
Facility	88,158,133	458.42 (26.90)	54.19 (6.09)	80.32 (7.10)	99.70 (13.09)	914.62 (34.19)	106.40 (12.46)	136.44 (10.92)	391.89 (15.32)
Person	50,914,148	499.82 (42.24)	79.36 (16.19)	105.00 (14.42)	114.99 (16.57)	1090.99 (40.77)	110.10 (15.39)	145.32 (12.35)	470.04 (20.45)
Person in facility	36,027,944	612.98 (54.67)	109.31 (24.75)	102.61 (9.60)	130.25 (13.98)	1181.32 (48.88)	124.56 (18.50)	162.08 (16.46)	500.49 (19.09)
Inapplicable/DK/Refused/Not Ascertained	56,042,866	253.24 (23.24)	39.31 (6.28)	70.67 (18.81)	30.02 (3.54)	544.34 (38.95)	79.40 (11.55)	84.67 (11.62)	177.70 (15.42)
MSA									
No	36,422,877	431.49 (44.95)	67.81 (14.96)	86.04 (10.90)	101.89 (21.91)	943.33 (38.66)	103.57 (11.33)	120.58 (14.05)	438.22 (19.20)
Yes	194,720,214	410.99 (16.35)	58.74 (5.20)	83.70 (7.87)	79.21 (6.21)	961.96 (26.29)	107.73 (8.69)	139.66 (7.78)	394.13 (10.66)
Census region					*	**	**		***
Northeast	42,648,623	348.84 (31.97)	45.16 (8.30)	62.16 (5.75)	63.91 (6.43)	816.17 (40.42)	69.79 (12.85)	111.04 (10.26)	342.83 (18.13)
Midwest	50,201,597	422.39 (31.59)	51.59 (6.46)	77.42 (9.14)	84.48 (9.90)	971.51 (36.22)	125.94 (16.51)	127.89 (11.71)	407.97 (15.99)
South	84,788,746	409.33 (25.42)	70.70 (10.41)	78.41 (7.90)	105.71 (14.70)	999.80 (42.84)	129.61 (14.21)	146.33 (10.47)	446.84 (17.67)
West	53,504,125	460.54 (37.30)	62.24 (11.16)	113.30 (26.14)	60.53 (6.82)	995.34 (53.21)	78.36 (14.42)	149.22 (18.05)	367.94 (19.11)
**Need factors**									
Perceived health status		***	**		***	***	**	*	***
Excellent/VG/Good	201,378,534	395.66 (14.58)	55.34 (4.84)	77.03 (5.52)	77.30 (6.28)	871.18 (22.03)	86.11 (6.84)	128.67 (7.52)	350.75 (9.11)
Fair/Poor	29,504,968	785.96 (112.00)	154.67 (35.60)	225.80 (91.73)	183.97 (26.39)	1297.49 (65.43)	187.49 (29.51)	165.58 (15.54)	600.73 (28.50)
Perceived mental health status		**			***	**			***
Excellent/VG/Good	213,789,726	397.55 (14.70)	58.32 (5.10)	78.08 (5.39)	76.49 (6.26)	928.97 (20.95)	103.18 (7.64)	136.39 (7.30)	379.55 (9.09)
Fair/Poor	17,045,611	820.96 (141.11)	102.25 (32.65)	231.23 (116.25)	226.00 (30.29)	1207.97 (85.47)	139.07 (31.46)	135.91 (12.63)	588.67 (42.91)
IADL help screener§					*	***			***
No	222,895,048	412.04 (15.29)	59.90 (5.03)	84.19 (7.57)	80.81 (6.11)	925.28 (23.07)	102.72 (7.91)	135.71 (6.93)	380.60 (8.55)
Yes	7,803,288	688.94 (165.46)	83.56 (46.33)	55.99 (14.10)	261.14 (71.71)	1507.95 (146.90)	176.97 (37.24)	145.75 (28.25)	754.43 (63.62)
ADL help screener				***		***			***
No	226,792,756	414.34 (15.30)	59.73 (4.99)	84.27 (7.54)	81.93 (6.10)	938.78 (22.22)	104.98 (7.92)	135.48 (6.77)	391.41 (8.79)
Yes	3,790,806	451.58 (163.29)	98.89 (50.39)	23.51 (6.57)	204.96 (109.83)	1654.05 (185.65)	173.56 (41.07)	164.66 (47.75)	776.80 (82.22)

*p <0.05, **p <0.01, ***p <0.001.

Table 4 shows multiple regression analyses results in looking at the relationship between total medical expenditures and the covariates included in our study. Individuals with one or more chronic conditions were found to spend $2,243 more on medical expenditures than those without chronic conditions, after controlling for other factors (p <0.001). Individuals with chronic conditions spent more on hospital care ($977, p <0.001), physician office visits ($326, p <0.001), and prescription drugs ($734, p <0.001) compared to those with no chronic conditions. With respect to age, holding all else constant, adults 18–64 experienced $2,157 less in total medical expenditures than adults 65 and over (p <0.001). A similar relationship is apparent in all expenditure categories (p <0.05). Men spent $383 less, on average, compared to women on medical expenditures (p <0.05).

**Table 4:** **Multivariate regression: medical expenditures and population characteristics**

	**Medical expenditure (Mean, SE)**			
	**Total medical expenditure**	**Total hospital expenditure**	**Total physician office expenditure**	**Total prescription drug expenditure**
**Predisposing factors**				
Chronic condition (ref: Without Chronic Conditions)	2243.09 (227.23)***	976.92 (188.93)***	326.39 (40.56)***	733.71 (57.1)***
Age in years (reference: Above 64)				
18-64	−2156.66 (368.11)***	−707.41 (281.64)*	−566.7 (106.35)***	−266.9 (112.87)*
Sex (reference: Female)				
Male	−383.35 (193.91)*	−61.17 (156.14)	−147.31 (52.41)**	−40.39 (49.49)
Race/Ethnicity (reference: Non-Hispanic White)				
Non-Hispanic Black	−149.98 (294.67)	248.36 (221.98)	−96 (61.35)	−254.7 (74.58)***
Hispanic	−659.51 (219.49)**	−317.77 (154.5)*	−39.56 (53.64)	−104.41 (91.06)
Non-Hispanic Asian	−1593.74 (252.59)***	−705.99 (204.49)***	−174.7 (94.64)	−395.28 (61.18)***
Others	−558.01 (627.92)	172.42 (575.5)	−206.19 (70.82)**	−243.07 (114.06)*
Health insurance (reference: Private)				
Public	−405.39 (395.6)	−224.58 (275.15)	−263.21 (84.74)**	219.26 (99.21)*
No insurance	−1731.07 (214.31)***	−726.95 (173.72)***	−348.4 (41.98)***	−361.19 (36.76)***
Highest education degree (reference: No Degree)				
High school diploma	947.26 (264.82)***	417.31 (205.75)*	204.41 (63.93)**	262.88 (79.29)**
Bachelor’s degree and above	1892.78 (374.73)***	897.43 (314.62)**	326.3 (65.4)***	440.92 (98.09)***
Other degree	1565.37 (455.16)***	1004.74 (409.8)*	249.62 (81.67)**	283.87 (119.72)*
Marital status (reference: Not Married)				
Married	442.78 (185.59)*	497.8 (145.61)***	32.94 (44.5)	25.23 (50.5)
Employment status(reference: Not employed)				
Employed	−952.34 (320.06)**	−122.56 (239.05)	−244.73 (71.24)***	−601.66 (79.81)***
**Enabling factors**				
**Income (reference: <$20,000)**				
$20,000-$39,999	132.18 (389.94)	−355.09 (333.57)	145.68 (64.16)*	131.49 (65.21)*
> = $40,000	−208.39 (294.82)	−460.79 (243.63)	79.43 (73.06)	125.02 (75.37)
Provider type of USC (reference: Facility)				
Person	−35.18 (259.88)	26.79 (205.54)	−49.15 (72.56)	44.81 (68.73)
Person in facility	741.92 (341.66)*	377.36 (248.16)	−9.47 (75.44)	244.49 (90.3)**
Inapplicable/DK/Refused/Not Ascertained	−983.66 (257.82)***	−72.43 (232.17)	−287.62 (56.24)***	−397.41 (45.83)***
MSA (reference: No)				
Yes	306.84 (225.58)	186.47 (173.41)	72.15 (56.55)	−6.29 (72.89)
Census region (reference: South)				
Northeast	331.13 (265.04)	306.38 (198.54)	19.17 (68.35)	−134.88 (80.02)
Midwest	692.42 (268.9)*	558.63 (227)*	31.37 (71.52)	−77.32 (62.61)
West	105.04 (205.91)	−47.07 (161.15)	−12.26 (66.52)	−180.18 (62.58)**
**Need factors**				
Perceived health status(reference: Fair/Poor)				
Excellent/VG/Good	−6483.91 (585.11)***	−3991.18 (432.82)***	−814.26 (101.39)***	−1226.6 (146.74)***
Perceived mental health status (reference: Fair/Poor)				
Excellent/VG/Good	−18.05 (512.5)	360.85 (432.12)	−70.45 (99.02)	−326.05 (139.01)*
IADL help screener (reference: No)				
Yes	7468.67 (1412.92)***	3880.29 (1148.71)***	175.8 (169.5)	1229.41 (351.3)***
ADL help screener (reference: No)				
Yes	8738.81 (2143.73)***	4908.5 (1836.08)**	102.19 (268.6)	927.15 (461.93)*

*p <0.05, **p <0.01, ***p <0.001.

In looking at differences by race/ethnicity, non-Hispanic blacks spent $255 less on prescription drugs than non-Hispanic whites (p <0.001). Hispanics experienced less spending on both overall medical (−$660, p <0.01) and hospital expenditures (−$318, p <0.05). Non-Hispanic Asians experienced lower spending on total medical, hospital, and prescription drug expenditures (p <0.001). Lastly, individuals of other race/ethnicity groups experienced less spending on physician office visits and prescription drugs (p <0.05).

Health insurance status was a significant predictor of medical spending across groups. Holding all else constant, individuals with no insurance spent significantly less than those with private insurance across all categories (p <0.001). Individuals with public insurance spent $263 less than those with private insurance on total physician office visits (p <0.01) and $219 more on prescription drugs (p <0.05). Across all expenditure groups, individuals with more education spent significantly more than those with no degree (p <0.05). The difference was highest for those with a Bachelor’s Degree and above (p <0.01). Married adults spent $443 more on total health and $498 more on hospital expenditures than those who were not married (p <0.05). In addition, being employed was associated with lower expenditures on total medical care, physician office visits, and prescription drug use, compared to being unemployed (p <0.01).

Individuals with an income between $20,000 and $39,999 experienced higher spending on physician office visits and prescription drugs, compared to those with income of less than $20,000 (p <0.05). Adults who reported a person in a facility as their USC provider spent $741 more on total medical expenditures and $245 more on prescription drugs than those who reported a facility as their USC (p <0.05 and p <0.01, respectively). While significant differences existed for the inapplicable category, it is not clear what these differences represent. Few significant differences existed by census region. Individuals in the Midwest spent more on total medical ($692) and hospital expenditures ($559) than those in the south, holding all else constant (p <0.05). No significant differences existed by MSA residence.

Individuals who reported excellent, very good, or good health status spent significantly less than those with fair or poor health (p <0.001). Differences ranged from $814 less on physician office visits, to $6,484 less on total medical expenditures (p <0.001). These differences were only apparent on prescription drug spending in the mental health category, with individuals who reported high mental health status spending $326 less on prescription drugs, on average, than those with fair or poor self reported mental health. The use of an IADL or ADL screener was associated with significantly higher spending in total medical, hospital, and prescription drug expenditure categories.

Table 5 shows multiple regression analysis in investigating the effect of population characteristics on out-of-pocket medical expenditure. The relationships here are similar to the total expenditure categories. Holding all else constant, individuals with chronic conditions spent $294 more on total out-of-pocket medical expenditures (p < .001), $27 more on out-of-pocket hospital expenditures (p < .01), $38 more on out-of-pocket physician office expenditures (p < .01), and $191 more on out-of-pocket prescription drug expenditures (p < .001) than those with no chronic conditions. Individuals who perceived their health to be excellent, very good, or good spent less, on average, than those who reported fair or poor health across all out-of-pocket expenditure categories (p <0.001). Among individuals who perceived their mental health to be excellent, very good, or good, lower spending was found in total out-of-pocket medical expenditures (−$171, p <0.05) and out-of-pocket prescription drug expenditures (−$96, p <0.01) compared to those with fair or poor self-reported mental health. After controlling for other factors, no significant findings were found for those who used IADL or ADL screeners, with the exception of higher average out-of-pocket prescription drug spending among those who used an IADL screener compared to those who did not ($176, p <0.05).

**Table 5:** **Multivariate regression: out-of -pocket medical expenditures and population characteristics**

	**Medical expenditure (Estimate, SE)**			
	**Total out-of-pocket medical expenditure**	**Out-of-pocket hospital expenditure**	**Out-of-pocket physician office expenditure**	**Out-of-pocket prescription drug expenditure**
**Predisposing factors**				
Chronic condition (ref: Without Chronic Conditions)	294.39 (25.15)***	26.67 (9.94)**	38.36 (12.14)**	190.64 (11.7)***
Age in years (reference: Above 64)				
18-64	−328.81 (49.38)***	18.87 (21.45)	9.72 (13.3)	−166.7 (25.89)***
Sex (reference: Female)				
Male	−158.87 (24)***	−11.45 (8.88)	−49.03 (9.21)***	−38.95 (10.63)***
Race/Ethnicity (reference: Non-Hispanic White)				
Non-Hispanic Black	−308.92 (30.25)***	−6.84 (19.75)	−67.9 (9.3)***	−105.82 (12.62)***
Hispanic	−234.85 (28.76)***	−21.26 (11.4)	−48.97 (13.9)***	−86.39 (12.54)***
Non-Hispanic Asian	−329.85 (37.67)***	−37.54 (11.1)***	−76.1 (14.86)***	−108.45 (14.85)***
Others	−173.66 (97.5)	60.48 (75.79)	−50.38 (25.36)*	−53.3 (39.15)
Health insurance (reference: Private)				
Public	−320.08 (41.62)***	−43.83 (17.78)*	−28.08 (16.22)	−85.45 (26.41)**
No insurance	−32.82 (34.52)	2.67 (12.87)	0.97 (15.45)	13.73 (15.43)
Highest education degree (reference: No Degree)				
High school diploma	171.82 (29.54)***	8.45 (14.06)	29.56 (10.46)**	45.77 (15.12)**
Bachelor’s degree and above	361.81 (39.5)***	28.99 (20.72)	90.1 (12.72)***	74.84 (18.67)***
Other degree	175.86 (51.53)***	32.8 (27.94)	49.32 (18.09)**	7.63 (17.68)
Marital Status (reference: Not Married)				
Married	54.44 (27.75)	27.58 (10.39)**	5.73 (9.59)	38.66 (12.39)**
Employment status(reference: Not employed)				
Employed	−177.15 (36.58)***	−0.21 (14.1)	5.48 (15.97)	−85.49 (16.97)***
**Enabling factors**				
**Income (reference: <$20,000)**				
$20,000-$39,999	110.5 (32.49)***	−12.89 (15.68)	16.32 (13.56)	12.42 (12.99)
> = $40,000	38.4 (31.18)	−1.36 (12.51)	−3.32 (9.72)	7.01 (11.74)
Provider type of USC (reference: Facility)				
Person	78.65 (35.46)*	13.73 (14.22)	16.55 (12.55)	29.48 (17.05)
Person in facility	145.41 (42.41)***	29.96 (16.53)	17.33 (13.85)	48.01 (15.84)**
Inapplicable/DK/Refused/Not Ascertained	−151.05 (29.52)***	−17.69 (8.93)*	−7.66 (17.73)	−89.52 (9.71)***
MSA (reference: No)				
Yes	33.36 (34.03)	8.26 (9.4)	9.83 (9.78)	−6.37 (16.62)
Census region (reference: South)				
Northeast	−186.6 (45.05)***	−43.62 (13.03)**	−37.13 (9.89)***	−96.89 (16.24)***
Midwest	−61.16 (36.88)	−12.86 (12.54)	−19.86 (10.35)	−50.37 (14.46)***
West	33.03 (41.6)	−24.08 (12.95)	21.11 (18.18)	−58.25 (15.67)***
**Need factors**				
Perceived health status (reference: Fair/Poor)				
Excellent/VG/Good	−421.17 (58.85)***	−114.64 (29.99)***	−78.58 (20.79)***	−171.79 (23.62)***
Perceived mental health status (reference: Fair/Poor)				
Excellent/VG/Good	−170.93 (67.43)*	16.51 (26.78)	−28.69 (30.2)	−96.18 (30.4)**
IADL help screener (reference: No)				
Yes	231.64 (145.56)	47.75 (44.76)	−10.58 (28.98)	175.84 (67.89)*
ADL help screener (reference: No)				
Yes	226.11 (160.59)	9.91 (48.95)	18.78 (45.88)	56.06 (87.93)

*p <0.05, **p <0.01, ***p <0.001.

## Discussion and conclusions

This study reveals the impact that chronic conditions have on total and out-of-pocket medical spending, as well as hospital, physician office visit, and prescription drug expenditures. Individuals with one or more chronic conditions are found to spend $2,243 more, on average, on total medical expenditures, $977 more on hospital stays, $326 more on physician office visits, and $734 more on prescription drugs compared to those with no chronic disease, after holding other factors constant (p < .001). This relationship persists for out-of-pocket spending, where those with chronic conditions spend, on average, $294 more on medical costs (p < .001), $27 more on hospital stays (p < .01), $38 more on office visits (p < .01), and $191 more on prescription drugs (p < .001). Having one or more chronic conditions is associated with significantly higher expenditures, even after controlling for important covariates. These findings build on previous literature that chronic conditions are associated with significantly increased expenditures among adults, with more recent, comprehensive expenditure data, as well as the inclusion of predisposing, enabling, and need covariates that appropriately control for confounding factors [10,11].

Even after accounting for predisposing, enabling, and need factors, including having chronic conditions, our research reveals stark disparities in total and out-of-pocket expenditures by race/ethnicity, age, sex, health insurance status, and education level. This finding is consistent with the literature of prevailing disparities across racial /ethnic and socioeconomic status groups [19,20]. When considering total expenditures, compared to non-Hispanic whites, non-Hispanic blacks spent $255 less on prescription drugs (p < .001), Hispanics spent $660 less on total medical costs (p < .01) and $318 less on hospital stays (p < .05). Asians spent $1594 less on medical costs, $706 less on hospital stays, and $395 less on prescription drugs, compared to non-Hispanic Whites (p < .001). These disparities in spending by race/ethnicity also existed for out-of-pocket expenditures, where Blacks, Hispanics, and Asians spent significantly less than Whites across all categories (p < .001). These findings are consistent with that of previous research, which has revealed that minorities experience lower spending than whites, even after controlling for socioeconomic factors [21]. Additional findings reveal lower total medical spending by young Americans compared to older Americans (p < .001), lower spending by men compared to women (p < .05), greater spending by those with private insurance compared to the uninsured (p < .001), and greater spending by those with higher education (p < .001).

The lower medical, hospital, physician office, and prescription drug spending experienced by minorities, the uninsured and publicly insured, and individuals with lower education reveal the need for research that incorporates more comprehensive access to care and need measures. Many of these individuals experience compounded vulnerabilities, in addition to having one or more chronic conditions, yet spend significantly less than their counterparts. This leads to the question of whether there is an issue of equitable access to care for vulnerable populations, or one of over-consumption on the part of the more privileged groups. Additional research exploring this issue and potential avenues for intervention are necessary to explore the full scope of our findings. Previous research has proposed the need to look further into these issues, as well as target resources towards reducing health care disparities among sicker individuals [21]. The role of Government is to improve health and health care for the population, particularly those most vulnerable. One way to accomplish this is to enact zoning and land-use laws that create healthier places for residents to live [22].

There are limitations associated with this study. We only considered one year of data for our analysis. This may limit our ability to assess trends that exist over the previous years. Second, there may be variables outside of the scope of MEPS that may better account for predisposing, enabling, and need factors. Unobserved social and cultural factors that we were unable to account for may also influence our research. Nevertheless, the strengths of our study outweigh these limitations, as our research has significant implications for improvements in health care quality and outcomes.

All in all, chronic conditions can often be prevented. Diet, exercise, and nutritional counseling have been shown to reduce chronic disease incidence [23]. Only through prevention and ongoing chronic disease care will we be able to reduce the costs associated with chronic conditions. The findings of our research suggest that chronic disease treatment and prevention efforts should be strengthened and targeted towards particularly vulnerable subgroups, including racial and ethnic minorities and the uninsured who are diagnosed with chronic conditions. Policies that influence that distribution of support and resources should consider that these dually vulnerable groups experience disparities in health care spending and interventions may be necessary to ensure adequate and affordable access to care for these populations. Regulations should also provide the targeted populations for physical activity and exercise, e.g., easy access to fitness clubs, and organized sporting activities. An examination of the political process is needed to include opportunities for chronic disease prevention [13] Initiatives increasing education and outreach are critical in limiting the incidence of diseases, and thus, the social and economic burden borne by society and individuals. In the pursuit of economic development, policymakers should also pay attention to the health and wellbeing, as well as their equitable distribution among the population [24].
